# Revised D-A-CH reference values for the intake of biotin

**DOI:** 10.1007/s00394-021-02756-0

**Published:** 2022-01-03

**Authors:** Alexandra Jungert, Sabine Ellinger, Bernhard Watzl, Margrit Richter, the German Nutrition Society (DGE)

**Affiliations:** 1 Interdisciplinary Research Center for Biosystems, Land Use and Nutrition (IFZ), Biometry and Population Genetics, Justus Liebig University, Heinrich-Buff-Ring 26, 35392 Giessen, Germany; 2 Department of Nutrition and Food Sciences, Human Nutrition, Rheinische Friedrich-Wilhelms-University Bonn, Meckenheimer Allee 166a, 53115 Bonn, Germany; 3 Department of Physiology and Biochemistry of Nutrition, Max Rubner-Institut, Federal Research Institute of Nutrition and Food, Haid-und-Neu-Str. 9, 76131 Karlsruhe, Germany; 4 Department of Science, German Nutrition Society, Godesberger Allee 136, 53175 Bonn, Germany

**Keywords:** Biotin, Reference values, Dietary intake

## Abstract

**Purpose:**

The reference values for biotin intake for Germany, Austria and Switzerland lead back to a report in 2000. Following a timely update process, they were revised in 2020.

**Methods:**

For infants aged 0 to < 4 months, adequate biotin supply via human milk was assumed and in consequence the reference value reflects the amount of biotin delivered by human milk. For infants aged 4 to < 12 months, biotin intake was extrapolated from the reference value for younger infants. Due to missing data on average requirement, the reference values for biotin intake for children, adolescents and adults were derived based on observed intake levels. The reference value for lactating women considered in addition biotin losses via human milk.

**Results:**

The reference value for biotin intake for infants aged 0 to < 4 months was set at 4 µg/day and for infants aged 4 to < 12 months at 6 µg/day. In children and adolescents, the reference values for biotin intake ranged from 20 µg/day in children 1 to < 4 years to 40 µg/day in youths 15 to < 19 years. For adults including pregnant women, 40 µg/day was derived as reference value for biotin intake. For lactating women, this value was set at 45 µg/day.

**Conclusions:**

As deficiency symptoms of biotin do not occur with a usual mixed diet and the average requirement cannot be determined, reference values for an adequate biotin intake for populations from Germany, Austria and Switzerland were derived from biotin intake levels assessed in population-based nutrition surveys.

**Supplementary Information:**

The online version contains supplementary material available at 10.1007/s00394-021-02756-0.

## Introduction

The reference values for biotin intake for Germany [D], Austria [A] and Switzerland [CH] lead back to a report in 2000 [1]. Following a timely update process, the reference values for biotin intake were recently revised and published in German language in autumn 2020 [2]. The present paper provides a summary of this publication.

Biotin is a water-soluble, sulfur-containing vitamin of the B-group [3] that acts as covalently bound coenzyme of acetyl-CoA carboxylase (ACC, two isoforms), pyruvate carboxylase (PC), β-methylcrotonyl-CoA carboxylase (MCC) and propionyl-CoA carboxylase (PCC) [4]. These enzymes are involved in the synthesis of fatty acids and cholesterol, in gluconeogenesis and in the catabolism of odd-chain fatty acids and branched amino acids (leucine, isoleucine, valine) as well as methionine and threonine [3, 4]. Through biotinylation of histones, biotin is involved in the regulation of gene expression, cell proliferation, repair of DNA damages [5] and in the stability of the chromatin structure [6].

Diet-related biotin deficiency is rare. An unbalanced diet with a high proportion of raw egg white [3, 7] or total parenteral nutrition without an adequate biotin supplementation may cause biotin deficiency [8–10]. Lifestyle factors may also favour biotin deficiency. Chronic alcohol abuse inhibits biotin absorption [11], while smoking probably promotes biotin degradation [12]. Since anticonvulsants impair intestinal absorption of biotin [13] and enhance biotin catabolism [14, 15], an increased biotin requirement during long-term antiepileptic therapy can be assumed. Congenital defects of biotinidase and carboxylases as well as inflammatory bowel disease may also cause functional biotin deficiency [11].

In general, clinical signs assigned to biotin deficiency are rather unspecific for biotin and may also be seen for other vitamins of the B-group. They comprise growth retardation, dermatological symptoms such as seborrheic dermatitis and alopecia, conjunctivitis [16] as well as neurological disorders like ataxia, lethargy and paraesthesia [11, 16]. Furthermore, an impairment of the humoral and cellular immune response due to biotin deficiency has been observed [3, 4, 17]. Clinical symptoms of biotinidase deficiency additionally include seizures, hypotension, metabolic ketoacidosis, hearing loss and optic atrophy leading to impaired vision [18–20]. As holocarboxylase synthetase is needed for the binding of biotin to apocarboxylases, holocarboxylase synthetase deficiency leads to metabolic ketoacidosis within the first week of life, accompanied by lethargy, muscular hypotension, vomiting and dermatological symptoms [21].

Adverse effects in humans due to high-dose biotin supplementation (up to 20 mg/day) have not been observed [3, 22]. The usual biotin intake via food and supplements does not represent a health risk to humans [23]. However, according to the Institute of Medicine (IOM) [24] and the European Food Safety Authority (EFSA) [23], data regarding adverse health effects of very high biotin intake are insufficient to derive a tolerable upper intake level.

The revised reference values for biotin intake are assumed 1) to prevent deficiency-related conditions in nearly all healthy individuals within the population, 2) to ensure optimal physiological and psychological performance and 3) to facilitate the formation of body reserves [2]. The D-A-CH reference values are divided into three categories: *recommended intake values*, *estimated values for an adequate intake* and *guiding values*. Information on the average requirement is necessary to derive *recommended intake values*, which – per definition – meet the requirement of approximately 98% of the population, stratified by sex and age (this term corresponds to Recommended Dietary Allowance of IOM and to Population Reference Intake of EFSA). In case of insufficient information on the average requirement, *estimated values for an adequate intake* are given, which are usually based on observed or experimentally assessed nutrient intake levels of apparently healthy, well-nourished subjects of a defined population group (this term corresponds to Adequate Intake of IOM and EFSA). The term “*estimated”* underlines the uncertainty of this methodological procedure. Nevertheless, *estimated values for an adequate intake* are expected to provide still an appropriate information for a sufficient and safe intake. *Guiding values* are given for non-essential components in food with regard to preventive effects, but also for components with a highly variable requirement due to many influencing factors preventing the derivation of *recommended intake values* or *estimated values for an adequate intake* [2, 25]. The reference values for biotin are *estimated values for an adequate intake* (see “Derivation of the reference values for biotin intake” and Table 1).

**Table 1:** Estimated values for an adequate intake of biotin

Age	Biotin
	(µg/day)
*Infants*	
0 to under 4 months	4
4 to under 12 months	6
*Children and adolescents*	
1 to under 4 years	20
4 to under 7 years	25
7 to under 10 years	25
10 to under 13 years	35
13 to under 15 years	35
15 to under 19 years	40
*Adults*	
19 to under 25 years	40
25 to under 51 years	40
51 to under 65 years	40
65 years and older	40
Pregnant women	40
Lactating women	45

### Biotin absorption and metabolism

In food, biotin is present in unbound (free) or protein-bound form; the latter is predominantly found in meat and cereals [4, 16]. Free biotin is almost completely absorbed [22, 26, 27]. Protein-bound biotin can be absorbed in free form which requires cleavage by gastrointestinal proteases and peptidases to biocytin (ε-*N*-biotyl-L-lysine) and biotinyl peptides [3] and subsequent release of biotin via biotinidase [3, 18]. Avidin, a glycoprotein in raw egg white, strongly binds to biotin, and thus inhibits intestinal biotin absorption [28]. Heat exposure denatures avidin in egg white, preventing the complex formation and thus allowing biotin absorption [16].

Biotin is mainly absorbed in the small intestine [26, 29] and to a smaller extent in the proximal colon [26]. The absorption rate is higher during conditions of biotin deficiency or low biotin intake compared to adequate biotin status or pharmacological doses of biotin supply [11, 30]. At present, no data are available on the average bioavailability of biotin from a usual mixed diet.

In addition to biotin intake via food and supplements, biotin synthesis by intestinal microbiota may contribute to cover the biotin requirement. However, its contribution to biotin supply is still unclear [11].

Biotin uptake by epithelial cells of the intestinal brush-border membrane is mediated through a saturable sodium-dependent carrier [31], which also transports other vitamins such as pantothenic acid, and thus is called *human sodium-dependent multivitamin transporter* (hSMVT) [3, 32]. During supplementation with biotin at pharmacological doses, absorption will mainly occur via passive diffusion [33].

Biotin is transported across the basolateral membrane by a sodium-independent carrier-mediated system [29]. More than 80% of biotin circulates freely in plasma [4, 34], whereas a small proportion is reversibly or covalently bound to proteins (e.g., biotinidase) [4, 18, 19, 35]. The valeric acid side chain of biotin can be covalently bound to a lysyl residue of apoenzymes by the holocarboxylase synthetase, resulting in the synthesis of active carboxylases [3, 36, 37]. Proteolytic degradation of carboxylases allows the recycling of biotin [19]. Renal reabsorption of biotin is regulated via hSMVT [38]. The liver is the primary storage organ [3]. At present, data on the average biotin content in the human body are lacking.

Biotin is excreted primary via urine. The hSMVT regulates the reabsorption of biotin depending on the biotin status, thus reducing renal excretion during biotin deficiency [38]. With regard to the total biotin amount in 24-h urine, biotin is primarily excreted unchanged (> 50%) and to a smaller extent as its metabolites bisnorbiotin (13–23%) and biotin sulfoxide (5–13%) [22]. A small proportion (< 2%) is excreted via faeces [3, 27].

## Assessment of biotin status

Biotin status is frequently determined via concentrations of biotin and its metabolites (e.g., bisnorbiotin, biotin sulfoxide) in serum/plasma/urine or through functional parameters.

Due to impaired enzyme activities, biotin deficiency leads to an increased urinary excretion of certain organic acids [39], particularly 3*-*hydroxyisovaleric acid (3-HIA) [39, 40] and 3-HIA-carnitine [28]. However, not all subjects develop an increase in 3-HIA concentration after an avidin-induced subclinical biotin depletion [41]. Biotin excretion in 24-h urine often decreases after an avidin-induced depletion [39, 40]. Nevertheless, it is not always possible to distinguish between subjects with and without biotin depletion [41]. As urinary bisnorbiotin and biotin sulfoxide concentrations show a non-linear decrease in the phase of depletion, their suitability as biomarkers of biotin status is limited [39, 40]. Serum concentrations of biotin and its metabolites are considered as non-sensitive parameters of biotin status because significant changes during avidin-induced depletion frequently do not occur [39].

Functional parameters of biotin status include activity changes in biotin-dependent enzymes (e.g., MCC and PCC) as well as altered biotinylation of MCC and PCC in lymphocytes [41]. In a randomised *cross-over* study including 16 healthy subjects, a reliable distinction between biotin depletion (induced by intake of raw egg white shakes) and adequate biotin status (induced by dietary biotin intake of at least 30 µg/day) was only possible by the biotinylation of lymphocytic MCC and PCC [41].

At present, no gold standard exists for the assessment of biotin status. Moreover, for the currently used biomarkers, no consistent assessment criteria are yet available which allow to classify the measured concentrations or changes in biomarker activities in a deficient, an insufficient or a sufficient biotin status. Thus, biotin status should be determined using a combination of biomarkers. Due to lack of data, it is unclear which combination of biomarkers is the most reliable to determine biotin status. In general, the combination of urinary biomarkers (e.g., 3-HIA) and functional parameters (e.g., degree of biotinylation of lymphocytic MCC and PCC) seems to be reasonable to define adequacy of biotin status.

In studies with healthy subjects, the serum concentrations of biotin ranged between 140 and 356 pmol/l and the serum concentrations of the metabolites ranged between 21 and 591 pmol/l for bisnorbiotin and between 0 and 126 pmol/l for biotin sulfoxide, respectively (*n* = 15) [39, 42]. The amount of biotin excreted in 24-h urine ranged between 18 and 77 nmol, while the amounts of the metabolites ranged between 11 and 39 nmol for bisnorbiotin, between 5 and 19 nmol for biotin sulfoxide and between 77 and 195 µmol for 3-HIA, respectively (*n* = 10) [39, 42]. In a more recent investigation, stored samples of control subjects (including also subjects from the former mentioned studies) without reported biotin supplementation were reassessed using improved methods for biomarker determination. According to that investigation, normal daily excretions of biomarkers in 24-h-urine were defined as follows: biotin between 19 and 62 nmol, bisnorbiotin between 12 and 54 nmol, biotin sulfoxides between 6 and 15 nmol (defined by the 10th percentile to the 90th percentile, *n* = 19) and 3-HIA between 39 and 150 μmol (defined by range, *n* = 17) [40].

However, the performance of frequently used bioassays is variable. Microbial assays failed to determine the concentrations of biotin metabolites, while avidin-binding assays frequently underestimate the concentrations of metabolites due to their lower binding affinity to avidin [27]. Avidin-binding assays calibrated with biotin may overestimate free biotin on one hand and may underestimate the total biotin concentration, which also accounts for biotin metabolites, on the other hand [43]. Therefore, a two-step procedure is recommended involving a separation of biotin and its metabolites by chromatographic methods followed by a quantification against reliable standards [27, 40].

## Derivation of the reference values for biotin intake

### Infants

The reference values for the intake of biotin for infants aged 0 to under 4 months were derived based on the biotin content of human milk, which is considered to supply the optimal amount of biotin for infants [44, 45]. In human milk, biotin is primarily present in free form [46], in concentrations underlying diurnal fluctuations [47]. The mean biotin content of mature human milk from healthy mothers apparently not consuming biotin supplements during lactation was 4.5 and 5.3 µg/l in studies from Finland (*n* = 36–200) [48] and Great Britain (*n* = 27) [49], respectively. Thus, an average content of 4.9 µg/l is supposed. Due to potential racial/ethnic disparities in human milk composition/intake, only studies from European countries were considered. In both studies, mature human milk from mothers of term infants, who were exclusively breastfed, was investigated via microbiological assays. Assuming an average daily intake of human milk of around 750 ml [44, 50–53], biotin intake in an exclusively breastfed infant is approximately 3.7 µg/day. Therefore, the *estimated value for an adequate intake* of biotin (for explanation of the used term see introduction) for infants aged 0 to under 4 months is set at 4 µg/day (Table 1).

For infants aged 4 to under 12 months, data on the average biotin requirement are lacking. Thus, the reference value for infants aged 0 to under 4 months is used to derive the reference value for infants aged 4 to under 12 months, taking into account the differences in the average body mass and the allometric exponent, thus leading to an *estimated value for an adequate intake* of biotin of 6 µg/day (Supplementary Table 1). As the requirement of B vitamins depends on the metabolically active body mass, an allometric exponent of 0.75 is assumed due to the association between body mass and metabolic rate according to Kleibers´ law [54, 55].

### Children and adolescents

No data from balance studies on biotin requirement are available for children and adolescents. Since deficiency symptoms with a usual mixed diet have not been observed so far, the average biotin intake of children and adolescents in German-speaking countries were used to derive *estimated values for an adequate intake* of biotin. This approach is in line with EFSA [56], which used the reported average biotin intakes among children and adolescents in European countries to set Adequate Intakes for biotin.

Data on biotin intake levels among children and adolescents from Germany and Austria [57–60] are presented in Table 2. No data are available from national examination surveys for children and adolescents from Switzerland. The median intake in some age groups is slightly higher than the reference values published by EFSA [56]. However, data on biotin content in food are frequently based on bioassays with limited specificity for biotin [61, 62]. Furthermore, data on biotin intake are sometimes only available as mean and not as median. The latter, however, reflects the intake level more appropriately than the mean value, considering the skewed distribution and outlying values. There is no evidence that the EFSA reference values are insufficient. In the light of this and in view of the wide range of biotin intake levels, the EFSA values [56] were accepted. When using the age groups the D-A-CH reference values are based upon [2], the resulting *estimated values for an adequate intake* of biotin range from 20 µg/day in children 1 to under 4 years old to 40 µg/day in youths 15 to under 19 years old (Table 1).

**Table 2:** Biotin intake among children and adolescents in Germany and Austria

Country	Age (years)	*n*		Biotin intake (µg/d)	
		Male	Female	Male	Female
*Germany*					
	1 to under 4^a^	242	246	28 (20–40)	25 (18–35)
	4 to under 5^a^	74	75	31 (22–42)	29 (21–41)
	6^b^	98	84	36 (18–65)	30 (17–53)
	7 to under 10^b^	313	274	37 (23–71)	35 (21–70)
	10 to under 12^b^	195	226	39 (22–65)	33 (19–64)
	12^b^	127	131	46 (29–261)	44 (22–136)
	13 to under 15^b^	222	244	49 (27–123)	41 (24–98)
	15 to under 18^b^	277	352	61 (29–172)	43 (23–108)
	15 to unter 19^c^	506	536	45 (42–48)	36 (34–37)
*Austria*					
	7 to under 10^d^	67	57	48 (24–73)	45 (34–56)
	10 to under 13^d^	83	81	43 (29–57)	30 (27–32)
	13 to under 15^d^	19	25	41 (13–68)	29 (25–33)

^a^Biotin intake among children in Germany obtained in the Consumption Survey of Food Intake among Infants and Young Children in Germany (2001–2002) via 3-day dietary records; data are presented as median [57] and 10th to 90th percentile [H Heseker 2013, personal communication, 28 January]

^b^Biotin intake among children and adolescents in Germany obtained in the nutrition module EsKiMo II of the German Health Interview and Examination Survey for Children and Adolescents (KiGGS) (2015–2017) via dietary records; data are presented as median and 10th to 90th percentile [58]

^c^Biotin intake among adolescents in Germany obtained in the National Nutrition Survey II (2005–2006) via two 24-h recalls; data are presented as median and 95 % CI-median [59]

^d^Biotin intake among children in Austria obtained in the Consumption Survey of Food Intake among Infants and Young Children in Germany (2010–2012) via 3-day dietary records; data are presented as mean and 95 % CI [60]

### Adults

Experimental studies investigating depletion and repletion phases at different dietary biotin intake levels are not available. In this context, it has to be considered that knowledge about dose–response-relationship is scarce and the biotin content of foods is only partially known [62]. Furthermore, data on the assessment of biotin status are insufficient (see “Assessment of biotin status”). Due to the resulting uncertainty in the determination of biotin requirement, only *estimated values for an adequate intake* of biotin can be derived. As symptoms of deficiency do not occur with a usual mixed diet, the *estimated values for an adequate intake* of biotin are based on the average intake in the general population, in line with the approach of EFSA [56].

Data on biotin intake levels among adults from Germany and Austria [59, 60, 63] are presented in Table 3. No data are available from national examination surveys for adults from Switzerland. There is no evidence to suggest gender-specific or age-related biotin reference values for adults. Consequently, the *estimated value for an adequate intake* of biotin was set to 40 µg/day for adults at the age of ≥ 19 years.

**Table 3:** Biotin intake among adults in Germany and Austria

Country	Age (years)	*n*		Biotin intake (µg/d)	
		Male	Female	Male	Female
*Germany*^a^					
	19 to under 25	469	486	46 (45–48)	39 (37–40)
	25 to under 35	614	852	48 (46–49)	42 (41–43)
	35 to under 51	1946	2648	48 (47–49)	41 (41–42)
	51 to under 65	1460	1740	47 (46–48)	41 (40–42)
	65 to 80	1165	1331	43 (42–44)	39 (38–40)
*Austria*					
	19 to under 25^b^	89	181	76 ± 75	55 ± 69
	25 to under 51^b^	478	856	63 ± 55	48 ± 38
	51 to under 65^b^	169	245	59 ± 51	46 ± 32
	65 to 80^c^	76	100	36 (33–40)	43 (34–53)

^a^Biotin intake among adults in Germany obtained in the National Nutrition Survey II (2005–2006) via two 24-h recalls; data are presented as median and 95 % CI-median [59]

^b^Biotin intake among adults in Austria obtained via two 24-h recalls (2014–2016); data are presented as mean and standard deviation [63]

^c^Biotin intake among adults in Austria obtained via two 24-h recalls (2010–2012); data are presented as mean and 95 % CI [60]

### Pregnancy

An increased renal 3-HIA excretion has been observed repeatedly during pregnancy [64–66], probably due to changes in glomerular filtration rate or to increased biotin turnover [4]. Results on other status parameters are inconsistent. Renal biotin excretion in pregnant women in the 3rd trimester was lower than in non-pregnant women in one study [43]. Another study did not show any differences in the excretion of biotin and bisnorbiotin between pregnant and non-pregnant women [66]. Since the clinical relevance of these observations remains unclear and deficiency symptoms have not been observed so far, neither in the foetus nor in the mother with a usual mixed diet, no additional requirement for pregnant compared with non-pregnant women is presumed. Thus, for pregnant women, the same intake values of biotin were considered to be adequate as for non-pregnant women.

### Lactation

Lactating women probably have an increased turnover and degradation of biotin [66], but clinical symptoms of deficiency have not been observed with a usual mixed diet comprising the diversity of foods (plant- and animal-based foods). A positive association between maternal plasma concentration and biotin concentration in human milk was reported, albeit the latter is much higher and the association was predominantly observed during the first months after delivery [48, 66]. Thus, one can speculate that maternal biotin intake also affects biotin concentration in human milk [47]. Experimental data on the biotin requirement of lactating women are not available. Considering an average biotin loss of approximately 4 µg/day due to lactation (see derivation for infants at the age of 0 to under 4 months), an *estimated value for an adequate intake* of biotin for lactating women of 45 µg/day is derived.

## Discussion

Due to the lack of sufficient data on average biotin requirement, the revised D-A-CH reference values for biotin are still *estimated values for an adequate intake*. Unlike before, the reference values for all age groups are now set as single values and not as ranges (Table 1). However, several uncertainties still exist with regard to specificity of analytical methods to determine biotin content in food.

Foods with a high biotin content include offal, like liver and kidney (cooked beef liver 121 µg/100 g, cooked veal kidney 81 µg/100 g), nuts (hazelnuts 62 µg/100 g, walnuts 36 µg/100 g), sunflower seeds (56 µg/100 g), eggs (25 µg/100 g), soybeans (23 µg/100 g), oatmeal (20 µg/100 g) and mushrooms (cooked champignon 15 µg/100 g, cooked chanterelle 14 µg/100 g). Due to their high consumption, milk and dairy products (cow’s milk 4 µg/100 g, cream cheese with at least 10% fat in dry matter 7 µg/100 g) also contribute to biotin supply [67].

Compared to the previous D-A-CH reference values on biotin, which represent intake ranges in most cases, the current reference values are at the upper end or above the previous ranges in children and at the lower end in infants ≥ 4 months, adolescents, adults and pregnant women. For infants aged 0 to under 4 months, the revised reference value is slightly lower than the former one. For lactating women, the average daily biotin loss due to breastfeeding has now been explicitly considered.

The *estimated value for an adequate intake* of biotin for infants aged 1 to under 4 years (20 µg/d) is more than three times higher than for the age group 4 to under 12 months (6 µg/d) (Table 1). That is in line with the Adequate Intake values for biotin set by EFSA (6 µg/d for the age group 7 to 11 months and 20 µg/d for children aged 1 to 3 years). Due to an age-related increase in fat-free mass [68], a physiological increase in biotin requirement is expected. However, the marked difference between these two reference values is predominantly explained by the different derivation approaches used to set the reference values for these age groups. For infants 4 to under 12 months, the reference value was extrapolated from the reference value for infants aged 0 to under 4 months using allometric scaling and reference values for body mass. For children 1 to under 4 years, the reference value was derived from median biotin intake values observed in this age group. Similar approaches were used by EFSA [56]. The food intake of children is considerably higher than the infant's food intake and the food consumed by children contains frequently higher amounts of biotin than human milk.

The current D-A-CH reference values on biotin intake for infants are almost congruent to the values of EFSA [56], IOM [69] and WHO [70], which were derived by a similar approach. For other age groups, the present reference values correspond to the reference values set by EFSA, whereas the D-A-CH reference values are considerably higher than the reference values set by IOM and WHO. However, it has to be considered that the age categories as well as the data basis of derivation differ among the authorities. While D-A-CH and EFSA reference values for subjects ≥ 1 years of age are based on observed biotin intake data, the adequate intake levels set by IOM and WHO are based on extrapolating the intake data from infants under consideration of allometric scaling. Overall, all three authorities derived Adequate Intakes because data on the average requirement are insufficient, what is in line with the present derivation approach. No authority assumed an additional biotin requirement for pregnant compared with non-pregnant women. Moreover, all authorities set higher reference values for lactating compared with non-lactating women due to the loss of biotin during lactation.

Reliable conclusions on the effects of biotin for the primary prevention of nutrition-related chronic diseases such as diabetes mellitus or neurological diseases cannot be drawn as randomised controlled trials on biotin supplementation for primary prevention of such diseases are lacking. Human intervention studies on the effect of biotin supplementation for secondary or tertiary prevention of diabetes mellitus or neurological diseases [71–75] frequently did not observe beneficial effects. However, in most of these studies, a distinction of the biotin effects from concomitant medication is not possible or high-dose biotin supplements were used. Hence, despite potential beneficial effects from biotin on glucose and lipid metabolism [76–81] observed in vitro and in animal studies (e.g., increased expression of glucokinase [78, 82], increased insulin secretion, improved glucose tolerance [78] and increased total beta-cell area relative to pancreas area [78, 79]), no preventive effects of biotin intakes exceeding the current reference values can be actually assumed with regard to nutrition-related chronic diseases. However, EFSA has approved several Article 13 health claims for biotin with regard to the “maintenance of normal skin and mucous membranes”, “maintenance of normal hair”, “contribution to normal psychological functions” and “normal macronutrient metabolism” [83].

## Conclusions

Since biotin requirement could not be determined, the revised D-A-CH reference values for an adequate biotin intake are based on the average biotin intake in the general population. This intake is considered to be sufficient to meet the requirements as symptoms of deficiency do not occur with a usual mixed diet. An adequate biotin supply can be ensured with a balanced diet including biotin-rich foods, such as eggs, nuts, oatmeal and mushrooms. Further research on the precise biotin content in food and on biotin requirement is warranted.

## Supplementary Information

Below is the link to the electronic supplementary material.

Supplementary file1 (DOCX 14 KB)
