# Correction to: Revised D-A-CH reference values for the intake of biotin

**DOI:** 10.1007/s00394-022-02824-z

**Published:** 2022-03-18

**Authors:** Alexandra Jungert, Sabine Ellinger, Bernhard Watzl, Margrit Richter, the German Nutrition Society (DGE)

**Affiliations:** 1 Interdisciplinary Research Center for Biosystems, Land Use and Nutrition (IFZ), Biometry and Population Genetics, Justus Liebig University, Heinrich-Buff-Ring 26, 35392 Giessen, Germany; 2 Department of Nutrition and Food Sciences, Human Nutrition, Rheinische Friedrich-Wilhelms-University Bonn, Meckenheimer Allee 166a, 53115 Bonn, Germany; 3 Department of Physiology and Biochemistry of Nutrition, Max Rubner-Institut, Federal Research Institute of Nutrition and Food, Haid-und-Neu-Str. 9, 76131 Karlsruhe, Germany; 4 Department of Science, German Nutrition Society, Godesberger Allee 136, 53175 Bonn, Germany

## Correction to: European Journal of Nutrition 10.1007/s00394-021-02756-0

The original version of this article unfortunately contained a mistake. In Tables 2 and 3, the footnote misses the “a, b, c, d” segmentation, so the letters in the table are not properly explained.

The corrected Tables 2 and 3 are given in the following pages:

**Table 2:** Biotin intake among children and adolescents in Germany and Austria

Country	Age (years)	*n*		Biotin intake (µg/d)	
		Male	Female	Male	Female
*Germany*					
	1 to under 4^a^	242	246	28 (20–40)	25 (18–35)
	4 to under 5^a^	74	75	31 (22–42)	29 (21–41)
	6^b^	98	84	36 (18–65)	30 (17–53)
	7 to under 10^b^	313	274	37 (23–71)	35 (21–70)
	10 to under 12^b^	195	226	39 (22–65)	33 (19–64)
	12^b^	127	131	46 (29–261)	44 (22–136)
	13 to under 15^b^	222	244	49 (27–123)	41 (24–98)
	15 to under 18^b^	277	352	61 (29–172)	43 (23–108)
	15 to unter 19^c^	506	536	45 (42–48)	36 (34–37)
*Austria*					
	7 to under 10^d^	67	57	48 (24–73)	45 (34–56)
	10 to under 13^d^	83	81	43 (29–57)	30 (27–32)
	13 to under 15^d^	19	25	41 (13–68)	29 (25–33)

^a^Biotin intake among children in Germany obtained in the Consumption Survey of Food Intake among Infants and Young Children in Germany (2001–2002) via 3-day dietary records; data are presented as median [57] and 10th to 90th percentile [H Heseker 2013, personal communication, 28 January]

^b^Biotin intake among children and adolescents in Germany obtained in the nutrition module EsKiMo II of the German Health Interview and Examination Survey for Children and Adolescents (KiGGS) (2015–2017) via dietary records; data are presented as median and 10th to 90th percentile [58]

^c^Biotin intake among adolescents in Germany obtained in the National Nutrition Survey II (2005–2006) via two 24-h recalls; data are presented as median and 95% CI-median [59]

^d^Biotin intake among children in Austria obtained in the Consumption Survey of Food Intake among Infants and Young Children in Germany (2010–2012) via 3-day dietary records; data are presented as mean and 95% CI [60]

**Table 3:** Biotin intake among adults in Germany and Austria

Country	Age (years)	*n*		Biotin intake (µg/d)	
		Male	Female	Male	Female
*Germany* ^a^					
	19 to under 25	469	486	46 (45–48)	39 (37–40)
	25 to under 35	614	852	48 (46–49)	42 (41–43)
	35 to under 51	1946	2648	48 (47–49)	41 (41–42)
	51 to under 65	1460	1740	47 (46–48)	41 (40–42)
	65 to 80	1165	1331	43 (42–44)	39 (38–40)
*Austria*					
	19 to under 25^b^	89	181	76 ± 75	55 ± 69
	25 to under 51^b^	478	856	63 ± 55	48 ± 38
	51 to under 65^b^	169	245	59 ± 51	46 ± 32
	65 to 80^c^	76	100	36 (33–40)	43 (34–53)

^a^Biotin intake among adults in Germany obtained in the National Nutrition Survey II (2005–2006) via two 24-h recalls; data are presented as median and 95% CI-median [59]

^b^Biotin intake among adults in Austria obtained via two 24-h recalls (2014–2016); data are presented as mean and standard deviation [63]

^c^Biotin intake among adults in Austria obtained via two 24-h recalls (2010–2012); data are presented as mean and 95% CI [60]

The original article has been corrected.

