# Supplementary material for: Revised D-A-CH reference values for the intake of biotin
Source: Eur J Nutr. 2022 Jan 3;61(4):1779–87. doi: 10.1007/s00394-021-02756-0 (PMC9106636; doi:10.1007/s00394-021-02756-0)
Supplement: Supplementary file 1 — Supplementary file1 (DOCX 14 KB) [file 394_2021_2756_MOESM1_ESM.docx]

**Supplementary Table 1** Derivation of reference values for biotin intake for infants from 4 to under 12 months

| Age (months) | Sex | Reference body mass ^a^  (kg) | Averaged biotin requirement considering reference body mass and allometric exponent ^b^  (µg/day) | Estimated intake for  biotin (rounded)  (µg/day) |
| --- | --- | --- | --- | --- |
| 4 to under 12 | male | 8.6 | 5.5 | 6 |
|  | female | 7.9 | 5.6 |  |

^a^ Reference values for body mass correspond to the median body mass of the WHO child growth standards [84] for the age of 8 months.

^b^ Calculated from: *estimated* *value*_0-4 months_ [µg/d] x (reference body mass_4-12 months_ [kg] / reference body mass_0-4 months_ [kg]) ^allometric exponent^; *estimated* *value*_0-4 months_: 4 µg/day (Table 1); reference body mass_0-4 months_: male 5.6 kg, female 5.1 kg [2] allometric exponent: 0.75
